# A new anti-inflammatory lupane in *Ziziphus jujuba* (L.) Gaertn. var. *hysudrica* Edgew.

**DOI:** 10.1016/j.heliyon.2024.e29989

**Published:** 2024-04-24

**Authors:** Dure-E Shahwar, N. Shehzadi, M Tanveer Khan, S. Zia, M. Saleem, S. Akhtar, Farhat Saghir, S. Iftikhar, A. Mobashar, S. Naheed, N.I. Bukhari, K. Hussain

**Affiliations:** a Punjab University College of Pharmacy, University of the Punjab, Lahore-54000, Pakistan; b Faculty of Pharmaceutical and Allied Health Sciences, Lahore College for Women University, Lahore, Pakistan; c Faculty of Pharmacy, The University of Lahore, Lahore, Pakistan

**Keywords:** *Ziziphus jujube***,***Rhamnaceae,* anti-inflammatory activity, Downregulation of inflammatory markers, Column chromatography, Hussainate

## Abstract

**Objectives:**

To investigate extracts of the stem bark of *Ziziphus jujuba* (L.) Gaertn. var. *hysudrica* Edgew. (*Rhamnaceae*) for anti-inflammatory activity and isolate the active principle(s).

**Methods:**

The dry powder was macerated separately in three types of solvents to prepare methanol extract (ME), ethyl acetate extract (EE), and chloroform extract (CE). Following in vitro anti-inflammatory screening, the most active extract was selected to isolate the active compound. Both, the active extract and isolated compound were further tested on rats using the carrageenan-induced inflammation model. The blood and paw tissue were subjected to qPCR, and histopathology, respectively.

**Key findings:**

CE showed comparatively higher anti-inflammatory activity (85.0–95.0 %) in all in vitro assays, except the heat-induced membrane stabilization model (*p* < 0.05), and upon column chromatography, it yielded a pure crystalline compound. The compound was a pentacyclic triterpenoid (Lupane), named as hydroxymethyl (3*β*)-3-methyl-lup-20(29)-en-28-oate (Hussainate). CE (500 mg/kg) and Hussainate (1.0 mg/kg) reduced edema in 5 h after carrageenan administration. The activity of Hussainate was found to be comparable to that of dexamethasone (standard). The possible activity mechanism was the downregulation of tumor necrosis factor-alpha (TNF-α), cyclooxygenase-2 (COX-II), NF-κB, and IL-1β.

**Conclusions:**

This study reveals that chloroform extract of the stem's bark of *Z. jujuba* may be used to prepare standardized anti-inflammatory herbal products using Hussainate as an active analytical marker. Hussainate may be used as a lead to develop anti-inflammatory drugs.

## 1 Introduction

Inflammation serves as a protective mechanism against physical injuries and infections, safeguarding the body from potential harm. Inflammation can progress into acute inflammation, and if left untreated, it can lead to chronic inflammatory diseases involving bones (Rheumatoid arthritis), lungs (Asthma and chronic bronchitis), kidneys (Glomerulonephritis), etc. On identifying inflammatory signals, mast cells, and macrophages promptly release pro-inflammatory cytokines, which then recruit leukocytes at the affected site and activate pathways involved in inflammation. The blood leukocytes route to the affected site with the primary objective of eliminating the root cause of inflammation while macrophages secrete pro-inflammatory mediators such as cyclooxygenase-2 (COX-II), interleukin-6 (IL-6), and tumor necrosis factor-alpha (TNF-α) to amplify the body's inflammatory response cascade [1].

Both acute and chronic inflammatory diseases are increasing day by day. Chronic inflammation is also associated with several comorbidities. Even in advanced countries such as the USA, diseases accompanying chronic inflammation are predicted to escalate continuously for the next 30 years [2]. This American population-based report indicated that 60% of the affected individuals had at least one, 42% had more than one, and 12% had five or more chronic inflammatory conditions. This indicated that chronic inflammatory diseases were among the foremost causes of global mortality [2]. Other inflammatory disorders like stroke, heart disease, chronic respiratory diseases, diabetes, obesity, and cancer were estimated to be responsible for three out of five fatalities [[3], [4], [5]].

The drugs to relieve inflammation are reported to reduce the risk of chronic inflammatory diseases [6]. There are several types of anti-inflammatory drugs available in the market to cope with inflammatory diseases [2]. However, the side effects of available anti-inflammatory drugs, gastric ulcers, nephrotoxicity, adverse cardiovascular events, hepatotoxicity, antiplatelet action, and anaphylactoid reactions involving the skin and pulmonary system, not only limit their use but necessitate to discovery of new anti-inflammatory molecules. Therefore, a traditionally medicinal plant has been selected to explore its anti-inflammatory potential and isolate active principle(s) which may serve as leads for drug development.

*Zizyphus jujube* (L.) Gaertn. var. *hysudrica* Edgew. belongs to the family *Rhamnaceae*. It is a hybrid plant produced from two species (*Zizyphus mauritiana* and *Zizyphus spina-christi*) [7]. The plant is tiny-thorny deciduous (5–12 m) and has lustrous green and ovate-acute leaves (Length 2–7 cm and width 1–3 cm). The leaf has 3 obvious veins at the base and a finely toothed margin. The flower is small (5 mm across) with 5 undetectable pale-green petals. The fruit is an edible, oval drupe (1.5–3.0 cm deep), smooth-green initially, brown to purplish-black, and wrinkled at ripening. The kernel is hard resembling an olive pit having two seeds.

The plant is well-known in Pakistan and many other countries for its oval-shaped large edible fruit (Seo Bair). Almost every part of the plant has traditional medicinal uses. The fruit and seeds are claimed to reduce stress in the Traditional Chinese and Korean Medicine Systems. The literature had several reports regarding the anti-inflammatory activities of members of the *Zizyphus* genus [[8], [9], [10], [11], [12], [13], [14]]. So, the plant under consideration is expected to have novel phytochemicals due to species-species hybridization, and anti-inflammatory activity similar to that of contributing plants. The literature contained a few reports regarding the isolation of cyclopeptide alkaloids from this species [[15], [16], [17], [18]] but there was no report regarding the isolation of anti-inflammatory principles. Therefore, this study focused on the isolation of anti-inflammatory compounds from the stem bark of the plant. This study may provide new leads to develop anti-inflammatory drugs and active markers to standardize extracts.

## 2 Results

### 2.1 Extraction

The maceration at room temperature was chosen to prepare extracts to avoid the effect of heat on phytoconstituents. The stem bark powder was extracted separately to get ethyl acetate extract (EE), chloroform extract (CE), and methanol extract (ME). Their yield was found to be 38.0, 38.0, and 108 mg/g, respectively. These results indicated that the yield of methanol extract was comparatively higher than that of other extracts. This is the evidence that the bark contained more polar contents.

### 2.2 Anti-inflammatory screening of extracts

**Fig. 1:**
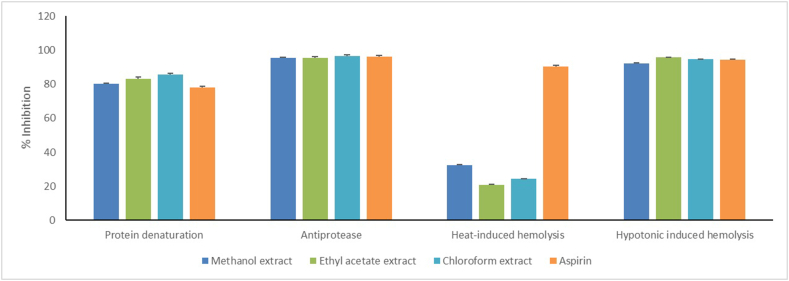
*In vitro* anti-inflammatory activity of extracts of stem bark of *Ziziphus jujube* (L.) Gaertn. var. *hysudrica* Edgew.

The anti-inflammatory response of three extracts determined using in vitro assays employing aspirin as a standard is given in Fig. 1. The inhibitory potential of ME, EE, and CE for heat-induced denaturation of protein was found to be 80.09, 83.09, and 77.90%, respectively. In this model, the activity of aspirin was found to be 77.94%. Likewise, antiprotease activities of ME, EE, CE, and aspirin were found to be 95.48, 95.56, 96.18 and 96.34%, respectively. It is noteworthy that in these in vitro models, the activities of all the extracts were comparable to the standard (aspirin). On the other hand, all extracts showed activities lower than that of the aspirin in protecting the RBC's membrane against heat-induced hemolysis (p < 0.05). However, in this assay, CE showed 24.35% membrane protection which was lesser than that of the ME but comparable to EE. In hypotonicity-induced RBC's membrane hemolysis, the activities of ES (17.18 %) and CE (12.55 %) were found to be lesser than that of the standard (52.46 %). As CE had been showing consistent anti-inflammatory behavior in all the in vitro assays, therefore, it was chosen for isolation.

### 2.3 Isolation

**Fig. 2:**
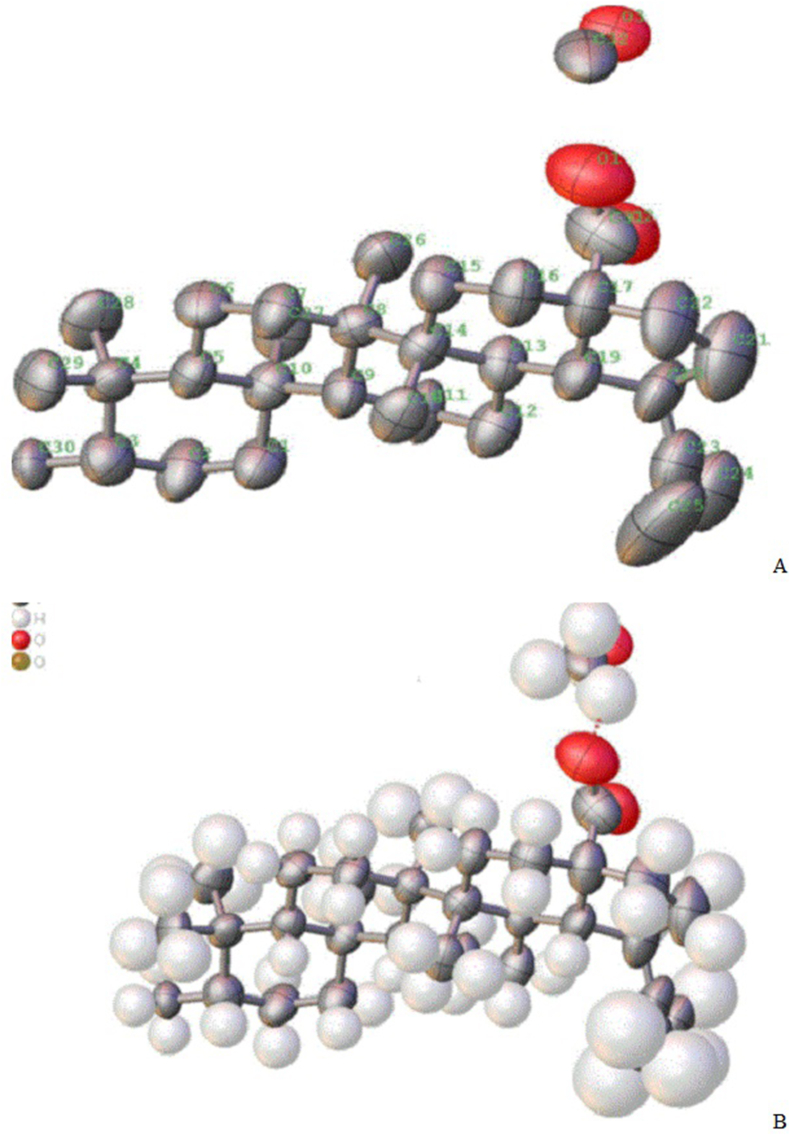
Carbon-oxygen and carbon-oxygen-hydrogen skeletons of hydroxymethyl (3*β*)-3-methyl-lup-20(29)-en-28-oate (Hussainate) A (Carbon-oxygen skeleton); B (Carbon-oxygen-hydrogen skeletons).

**Fig. 3:**
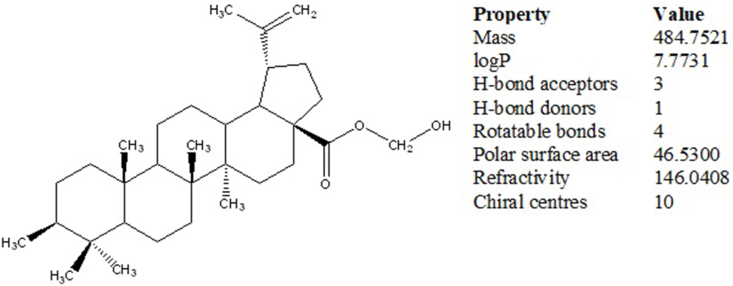
Chemical structure and predicted molecular properties of hydroxymethyl (3*β*)-3-methyl-lup-20(29)-en-28-oate (Hussainate).

**Fig. 4:**
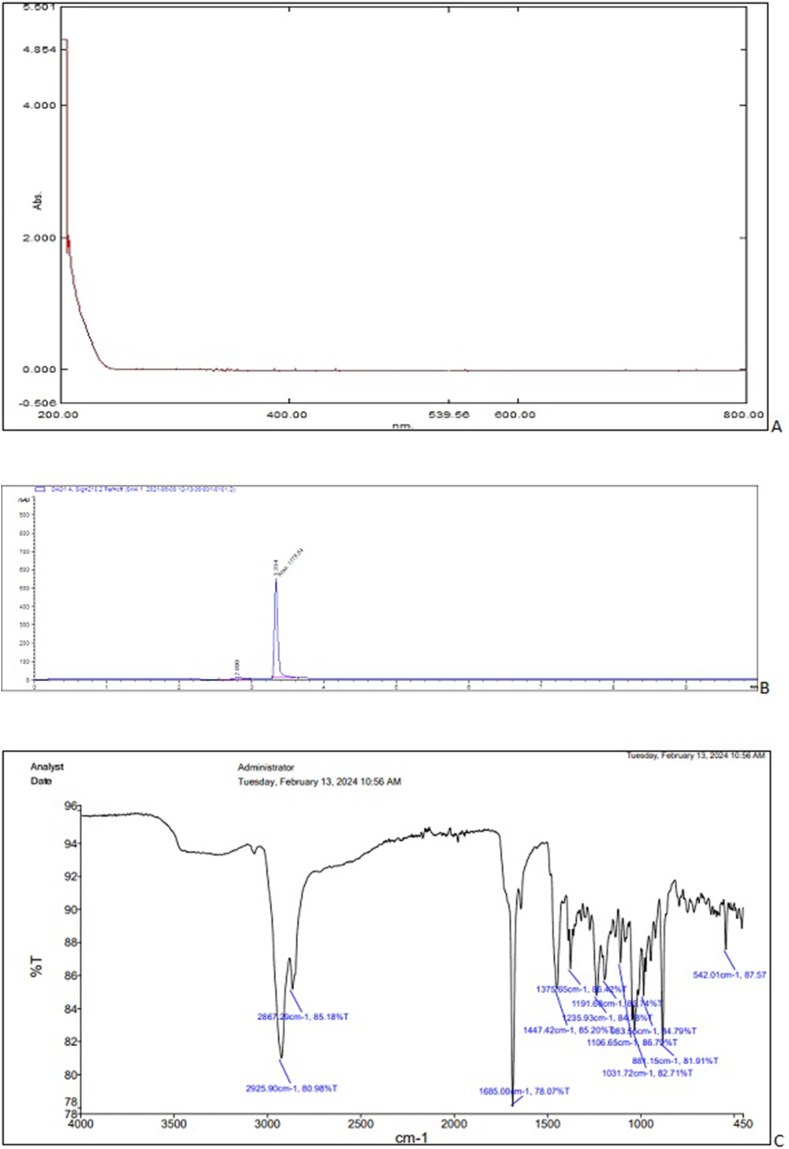
UV/Visible spectrum (A), HPLC chromatogram (B) and FTIR spectrum (C) of hydroxymethyl (3*β*)-3-methyl-lup-20(29)-en-28-oate (Hussainate).

**Fig. 5:**
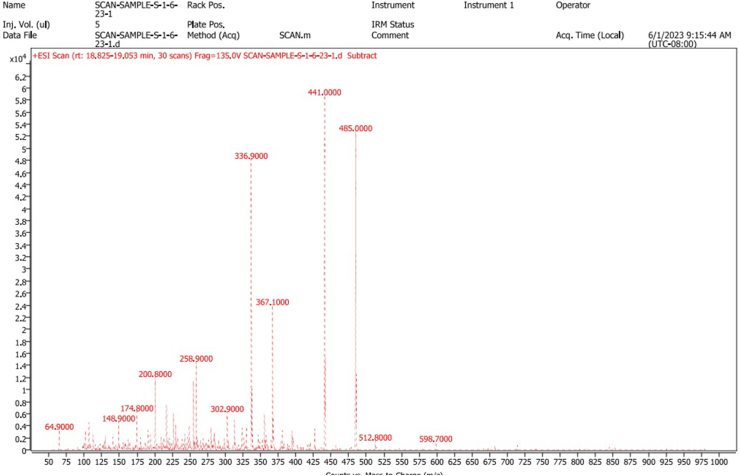
Mass spectrum of hydroxymethyl (3*β*)-3-methyl-lup-20(29)-en-28-oate (Hussainate).

**Fig. 6:**
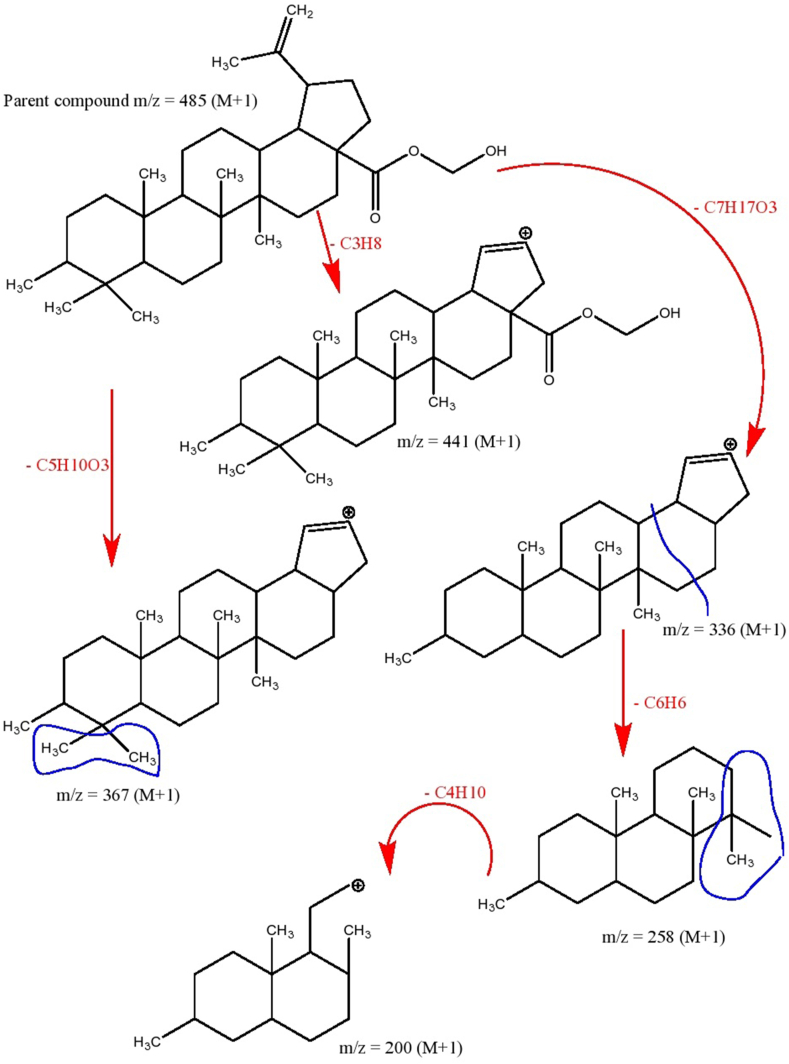
Fragmentation pattern of hydroxymethyl (3*β*)-3-methyl-lup-20(29)-en-28-oate (Hussainate).

The above-selected extract upon elution through a column yielded orthorhombic crystals. The crystals showed positive results for the Liebermann-Burchard and anisaldehyde reagent tests which indicated that the isolated compound was a pentacyclic triterpenoid. The X-ray diffraction crystallography confirmed the structure of the compound. The carbon-oxygen skeleton and chemical structure showing carbon, hydrogen, and oxygen atoms are given in Fig. 2 A & B, respectively. The chemical structure showing absolute configuration and some physical properties predicted using the Mcule Property Calculator are given in Fig. 3. The spectra (UV/Visible and IR) and HPLC chromatogram of the compound are given in Fig. 4 A, C & B, respectively. The UV/Visible spectrum showed that the compound in methanol absorbed a maximum at 210 nm (Fig. 4A). A single peak at T_R_ 3.334 min in HPLC chromatogram obtained eluting the sample through C18 column using phosphoric acid acidified water (pH 2.5) indicated the purity of the compound (Fig. 4B). IR spectrum (Fig. 4C) showed band of OH stretching (3350-3450 cm^−1^), aliphatic CH and CH_2_ stretching (2925.90 and 2869.29 cm^−1^), C=O stretching carbonyl (1685.12 cm^−1^), CH bending aliphatic (1447.29 cm^−1^), CH3 surface bending (1375.65 cm^−1^), C–O stretching (1191.68 cm^−1^). The ESI scan in positive mode (Fig. 5) showed a molecular ion peak [M+1] at *m*/*z* ratio at 485 corresponding to molecular formula C_32_H_52_O_3_ and a base peak at 441 corresponding to C_29_H_44_O_3_. A complete fragmentation pattern is given in Fig. 6. All the spectral data confirmed that the isolated compound was a lupane analogous to methyl betulinate. The compound was named as hydroxymethyl (3*β*)-3-methyl-lup-20(29)-en-28-oate or 3*β*-methyl-lup-20(29)-en-28-oic acid hydroxy methyl ester (Hussainate). The compound's structural features comparison to those reported earlier indicate that it is a new and the first report in *Z. jujuba*. Chemically, it seems to be a derivative of methyl betulinate, wherein the (CH_3_) is replaced by a hydroxy methyl moiety, and the hydroxyl group at C_3_ of the ring A is replaced by a methyl group. The presence of a methyl group at C_3_ is very unusual as most of such compounds are either unsubstituted at this position or have free/esterified OH or oxygen as keto (=O) or epoxide (-*O*-). During biosynthesis, oxygen is added as epoxide which is then reduced to hydroxyl group. C_3_ methylation may occur due to two reactions; the removal of protonated OH as H_2_O leaving electron-deficient carbon and a methyl radical attack or addition of CH_3_ and then the removal of H_2_O resulting in an unsaturation between C_2_ and C_3_, and reduction of this bond.

### 2.4 Anti-inflammatory activity (*In vivo* model)

**Fig. 7:**
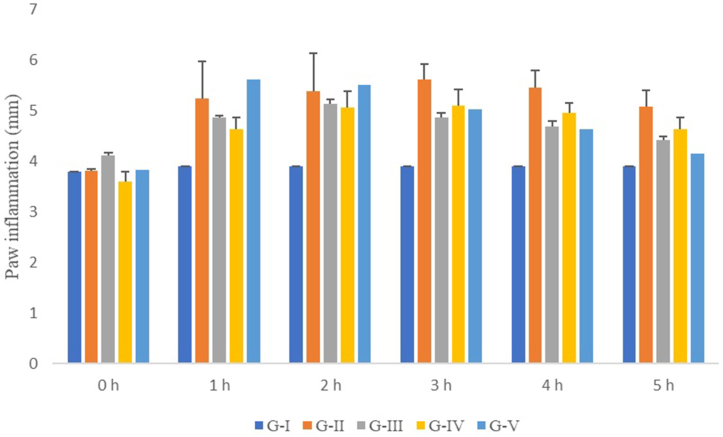
*In-vivo* anti-inflammatory effect using carrageenan-induced inflammatory model G-I (Group-I, normal control); G-II (Group-II, disease control); G-III (Group-III, dexamethasone-treated); G-IV (Group-IV, chloroform extract-treated); G-V (Group-V, Hussainate-treated).

Fig. 7 depicts the findings of the anti-inflammatory activity of CE and Hussainate utilizing the carrageenan-induced inflammatory assay. The animals in groups III (dexamethasone-treated) and IV (CE-treated) showed a rapid onset of anti-inflammatory response. In these groups, inflammation started decreasing from the first hour of the study, whereas, the animals of group V (Hussainate) exhibited comparatively a delayed response, the effect began after 3 h. In this study, the activity of dexamethasone, chloroform extract, and Hussainate was discovered to be comparable with each other. The activity of chloroform extract, comparable to the standard, indicated that it might contain multiple anti-inflammatory compounds. The disease control group (Group II) began to show inflammation reduction in the fifth hour of the study.

**Fig. 8:**
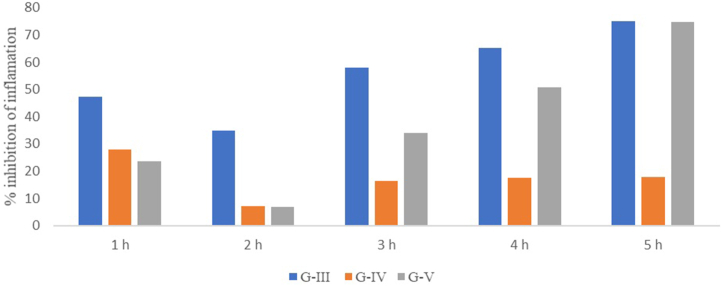
Inhibition of inflammation using the carrageenan-induced inflammatory model G-I (Group-I, normal control); G-II (Group-II, disease control); G-III (Group-III, dexamethasone treated); G-IV (Group-IV, chloroform extract-treated); G-V (Group-V, Hussainate-treated).

The outcomes of inflammation reduction noted for groups III to V are depicted in Fig. 8. In group III, inflammation reduction increased steadily from 1st to 5th h. The animals of group IV showed almost similar results as for the standard. Contrarily, the animals of group V began to show inflammation reduction at 3rd hour which was then continuously increased till the 5th hour of the study. The oral administration of CE showed a reduction in the paw edema till 5 h. The paw edema in the first 5 h for the CE-treated rats was 3.6, 4.63, 5.06, 5.10, 4.95, and 6.64 mm. Conversely, the paw edema in Hussainate-treated rats was 3.83, 5.61, 5.52, 5.02, 4.64 and 4.15 mm.

**Fig. 9:**
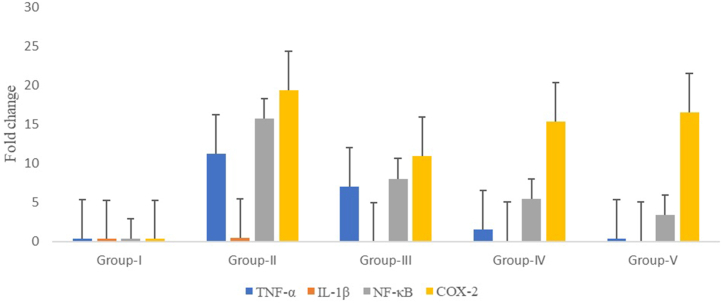
Inflammatory markers in blood samples by qPCR technique G-I (Group-I, normal control); G-II (Group-II, disease control); G-III (Group-III, dexamethasone-treated); G-IV (Group-IV, chloroform extract-treated); G-V (Group-V, Hussainate-treated).

The estimated inflammatory biomarkers of rats of different groups are shown in Fig. 9. These findings demonstrated that in comparison to group III, the downregulation of TNF-α was significantly higher in group IV and group V. The TNF-α downregulating effect of group V was similar to group I. The downregulation of IL-1β was almost similar in group-III, -IV, and -V. The downregulation of NF-κB was the same in group-II and -IV. The group-III had shown a higher tendency of COX-II downregulation as compared to that of the group-IV and -V (*p* < 0.05).

**Fig. 10:**
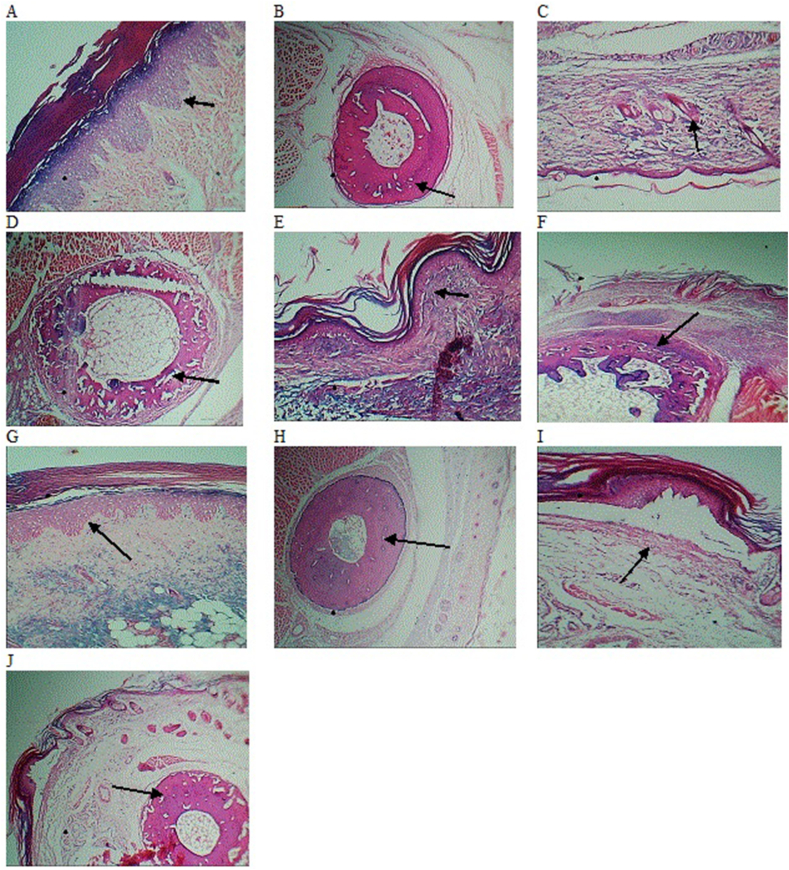
Images of histopathology of the paw tissue A-B (*Group-1, normal control)*, C-D (*Group-II, disease control)*, E-F (*Group-III, dexamethasone-treated)*, G-H (*Group-IV, chloroform extract-treated*), I-J (*Group-V, Hussainate-treated)*.

The histopathological features of the rat paw tissue of different groups (Fig. 10) indicated normal epidermal and dermal layers' structure of paw sections of group I with no evidence of mononuclear cell infiltration (Fig. 10A and B). On the other hand, acute edematous and pannus formation was observed in the disease control, group II (Fig. 10C and D). The paw tissue of dexamethasone-treated rats (Group III) showed less edematous and pannus formation as compared to group III (Fig. 10E and F). The CE-treated and Hussainate-treated rats (Group IV and -V) showed little infiltration and no pannus development (Fig. 10 G-H and Fig. 10 I-J, respectively). These results augmented the claim of anti-inflammatory effects of chloroform extract and Hussainate, obvious in the expression of biomarkers. According to the findings of the present investigation, oral administration of 500 mg/kg *Z. jujuba* stem bark's CE*,* and 1.0 mg/kg Hussainate possess good anti-inflammatory activity.

These findings prove the hypothesis that the selected hybrid plant just like other members of the genus *Ziziphus* has anti-inflammatory potential [19,20]. The activity of CE was not only higher amongst the extracts but also consistent in all the in vitro models. Hence, CE was selected for the isolation of the bioactive compound. The extract upon isolation gave a pentacyclic triterpenoid which was a new compound, structurally related to methyl betulinate. This compound has not been reported earlier in the genus *Ziziphus*.

Pentacyclic triterpenoids are secondary metabolites in many plants and show diverse pharmacological activities including anti-inflammatory, antiulcerogenic, hepatoprotective, anti-tumor, and anti-hypertensive activities [21]. Betulinic acid is reported in many species of the genus *Ziziphus* including contributing plants of the hybrid species under investigation [22,23]. Being a pentacyclic triterpenoid, the isolated compound was expected to have anti-inflammatory activity that was explored and compared with dexamethasone as a standard. The dose of Hussainate was selected based on its structural similarity to betulinic acid. The findings of the current study were consistent with the previous reports wherein steroids had shown anti-edematous response by preventing the pro-inflammatory cytokines (COX-II, iNOS and TNF-α) [24]. As expected from steroidal structural similarity, Hussainate showed good anti-inflammatory effects.

## 3 Discussion

The denaturation of protein has long been associated with the pathogenesis of inflammatory diseases. An injury to the tissue due to microbes, physical or chemical factors can lead to denaturation of the cellular or intracellular proteins which leads to the expression of antigens associated with type-III hypersensitivity reactions (serum sickness and glomerulonephritis) [25]. Heat-denatured proteins are native macromolecules that are very effective in initiating delayed hypersensitivity [26]. Recent reports reveal that phenylbutazone and indomethacin besides COX inhibition prevent protein denaturation as a part of their anti-inflammatory action [27]. Hence, the inhibitors of protein denaturation could be potential anti-inflammatory agents. So, this assay has been selected to find the anti-inflammatory activity of extracts. In the present study, all the extracts have prevented the denaturation of egg albumin, however, the activity of the chloroform extract was higher among the other extracts.

Proteases – serine proteases, cysteine proteases, and metalloproteases – are a large class of enzymes that hydrolyze the peptide linkage. These are involved in cancer, thrombosis, and inflammatory disorders [28,29]. These enzymes promote inflammatory responses by controlling the expression and activity of pro-inflammatory cytokines, chemokines, and other miscellaneous immune components. Thus, inhibition of proteases can prevent inflammatory conditions. In the present study, we have used trypsin which is a serine protease to find the anti-inflammatory potential of different extracts. All the extracts have shown excellent inhibitory potential for trypsin and prevented the hydrolysis of casein. Among the extracts, CE has shown higher proteinase activity.

Hemolysis of cell membranes occurs upon the accumulation of excessive fluids. The injured membrane increases the vulnerability of the cells to secondary damage due to peroxidation of lipids [30]. The lytic enzymes and inflammatory mediators released in response to an injury increase vasodilation, and vascular permeability, hence promoting blood flow and causing plasma exudation leading to edema. The leakage of serum proteins and intracellular fluids into the tissue can be prevented by stabilizing membranes. In the membrane stabilization assays, heat and hypotonic medium were used to bring about membrane lysis. The extracts were found to be very efficient in preventing the lysis of the RBC membrane, hence, they could prevent the release of lytic enzymes and inflammatory mediators. As observed earlier, chloroform extract has shown higher activity among the extracts. Based on the activity of CE in all the used anti-inflammatory assays, the isolation studies were performed on CE.

Carrageenan was used to induce edema in the paws of experimental animals. Carrageenan-induced paw edema assay, owing to its sensitivity, validity, reproducibility, and applicability in finding drugs active on mediators of acute inflammation. This method is often used for the determination of anti-inflammatory activity. The carrageenan inflammatory mechanism has two phases. Phase-I (neurogenic phase) involves the secretion of bradykinins, histamine, serotonin, and substance-P while phase-II (inflammatory phase) includes the release of prostaglandins and cytokines. Opioids can reverse the neurogenic phase whereas other anti-inflammatory drugs act in the inflammatory phase [31]. Both the chloroform extract and Hussainate inhibited the release of cytokines which indicated that they had acted in phase II of inflammation.

In this study, CE and Hussainate demonstrated a retrograding of IL-1β levels that indicated their inhibitory effects on pro-inflammatory mediator release. This marker plays a crucial role in cartilage breakdown and T-cell activation due to the generation of reactive oxygen species and proteolytic enzymes [32]. Hence, the extract and isolated compound are expected to be effective in treating the inflammation of cartilage. In RA, the production of prostaglandins (PGE2) is increased by COX-II. PGE2 subsequently acts on chondrocytes and encourages the release of TNF-α and IL-1β resulting in inflammation and discomfort. TNF-α and other inflammatory mediators along with adhesion molecules stimulate, both in an autocrine and paracrine manner, and cause rheumatoid synovitis [33]. According to qPCR expression analysis, the isolated compound, and chloroform extract downregulated the level of COX-II. So, these could be useful in reducing polygenetic arthritis symptoms. Histopathological microscopic examination of paw tissue also confirmed the role of the extract and isolated compound in minimizing inflammation and pannus formation. *In vitro* and *in vivo* data using multiple models confirm the anti-inflammatory potential of CE of the bark of the plant. CE and Hussainate downregulate the expression of COX-II, TNF-α, NF-kB, and IL-1β. Keeping these points in view, CE may be developed into a standardized herbal anti-inflammatory medicine. Moreover, Hussainate could be a potential anti-inflammatory drug candidate.

The anti-inflammatory effect of CE that is equivalent to that of Hussainate and dexamethasone indicates that CE contains other phytoconstituents that produce a synergistic action along with the isolated compound. These results indicate the need to explore chloroform extract of the plant for other active principles.

## 4 Conclusions

The chloroform extract from *Z. jujuba* stem bark shows great anti-inflammatory potential by preserving protein function, reducing protease-induced damage, and stabilizing cell membranes. In a carrageenan biphasic model, it effectively inhibits both early histamine and serotonin release and later prostaglandin and cytokine production. Additionally, the Hussainate exhibits immunomodulatory effects by downregulating mRNA expression of key pro-inflammatory cytokines such as TNF-α, IL-1β, IL-6, and COX II. These findings strongly recommend Hussainate as a valuable lead to developing anti-inflammatory drugs, and an active analytical standard. Chloroform extract of the stem bark of the plant may be used to develop an evidence-based anti-inflammatory herbal medicine. Moreover, Hussainate may be used to maintain constancy among batches.

## 5 Material and methods

### 5.1 Plant material

The stem's bark of the plant was gathered in the month of March 2015. Prof. Dr. Zaheer-u-Din Khan, Department of Botany, Govt. College University (GCU), Lahore, Pakistan, identified the plant. A voucher specimen bearing number 3571 was deposited in the herbarium of the GCU. The shade-dried material was pulverized.

### 5.2 Chemicals and solvents

The chemicals and solvents used included Tris HCl, sodium phosphate, potassium persulfate, carrageenan, disodium hydrogen phosphate, casein, sodium dihydrogen phosphate trypsin, sodium chloride and perchloric acid (Sigma-Aldrich, Germany), methanol, hexane chloroform, propylene glycol and gum acacia (Merck, Germany), aspirin (Highnoon Laboratories Limited, Pakistan), dexamethasone (Remington Laboratories, Lahore, Pakistan), ethyl acetate (Daejung, Korea), and normal saline (Surge Laboratories, Sheikhupura, Pakistan).

### 5.3 Instruments

Water bath (Thermo Fischer Scientific, USA), incubator (MIR-153), centrifuge (ScieNovo Lab, China), Model-2550 UV/Visible spectrophotometer (Shimadzu Corporation, Kyoto, Japan), Bruker D8 single crystal X-ray diffractometer equipped with high-efficiency photon II detector, qPCR, SYBR select master mix and QuantStudio3 real-time PCR (Applied Biosystems Thermo Scientific, USA).

### 5.4 Extraction

For 7 days, at room temperature, the powder material (500 g) was macerated separately in 2 L solvents such as methanol, ethyl acetate, and chloroform. The filtrates were dried under reduced pressure at 40 °C.

### 5.5 *In vitro* anti-inflammatory screening

#### 5.5.1 Heat-induced protein denaturation activity

The extract and standard (Aspirin) solutions of 100.0 μg/mL were prepared in Tris HCl buffer saline (TBS, 0.05 M, pH 6.80). The activity was measured using the reported method [34]. The test sample/standard solution (1.0 mL) was added into a test tube already having 5.0 mL 0.20 %, *w/v* egg albumin in TBS. A control was made in the same manner as the sample by substituting the volume of the test sample with TBS. The tubes containing the control, test sample, and standard were incubated for 20 min at room temperature. The test tubes were then heated in an oven at 70 ± 5 °C for 5 min and immediately cooled in ice-chilled water to prevent the denaturation of albumin. The resulting mixtures were analyzed in triplicate using a spectrophotometer at 660 nm. TBS was used as a blank and Equation (1) was used to determine activity.

(1) $$\text{Activity}\;\left(\%\right)=\left(\frac{A_{\text{Control}}-A_{\text{Sample}}}{A_{\text{Control}}}\right)\times 100$$

#### 5.5.2 Antiprotease activity

The sample and standard solutions used above were used to determine antiprotease activity using the reported method [35]. One milliliter each of trypsin (0.06 mg/mL), 20 mM Tris HCl buffer, and a test sample/standard were mixed in a test tube. A control was made with all of the components except the sample. The test sample and control were incubated at 37 ± 1 °C for 10 min. Then, 1.0 mL casein solution (0.80 % *w/v*) was mixed with the test solution, and the mixture was further incubated for 20 min. Finally, after adding perchloric acid (2.0 mL of 70 %, *v/v*), the test tubes were centrifuged at 2500 rpm for 10 min. The supernatant obtained was analyzed in triplicate at 210 nm using a blank comprising Tris HCl buffer. The same procedure was carried out with aspirin and control. The antiprotease activity was determined using Equation (1).

#### 5.5.3 Heat-induced hemolysis activity

Horse blood (3.0 mL) was obtained from the Hospital of the University of Veterinary and Animal Science, Lahore. The blood was centrifuged at 3000 rpm for 10 min to collect RBC cell-pellet that was mixed with 3.0 mL normal saline. The suspension was diluted using 40 % *v/v,* with isotonic phosphate buffer solution (IPBS). The extract and standard (Aspirin) solutions of 100 μg/mL concentration were prepared in IPBS. A reaction mixture comprising 1.0 mL of test sample or standard, 0.10 mL RBC suspension, and 4 mL IPBS was then incubated for 20 min at room temperature. The mixture was heated at 54 ± 2 °C for 20 min in the water bath to induce hemolysis. The contents were allowed to cool immediately and centrifuged at 1300 g for 5 min. The amount of hemoglobin released in the supernatant was estimated by analyzing samples in triplicate at 540 nm against IPBS as a blank. A control comprising all the ingredients, except the sample was treated as mentioned above. The activity was determined using Equation (1).

#### 5.5.4 Hypotonicity-induced hemolysis activity

The sample and standard solutions prepared as mentioned above were used in this model. The test mixture was prepared from 1.0 mL test sample/standard, 2.0 mL hyposaline (0.36 % *w/v*), 2.0 mL IPBS, and RBC solution (0.10 mL). The test mixture was kept at room temperature for 20 min, and centrifuged at 1300 g for 5 min. The absorbance of the supernatant was noted at 540 nm against IPBS as a blank. Equation (1) was used to calculate the activity.

#### 5.5.5 Isolation studies

Silica gel 70 g (Kiesel gel 60H) was packed into a glass column (40 × 3 cm). CE (15 g) was dissolved in 10 mL chloroform. The sample was loaded on the adsorbent, and eluted with a 100 mL mixture of hexane and dichloromethane (Starting with pure hexane and then the mixture of hexane and dichloromethane, every time a 10% decrease in hexane). It produced 22 fractions, out of which fractions 8, 9, and 10 were pooled based on similar TLC profiles. The solvent was evaporated resulting in 1.73 g FA that was again eluted through a glass column, 10 × 1.5 cm, packed/filled with 20 g silica gel. The elution was carried out using a 10 mL solvent mixture composed of ethyl acetate, hexane, and methanol (starting with 8:1:1, *v/v/v* and ending at 1:8:1, *v/v/v*). The resulting eight sub-fractions were kept at room temperature for slow evaporation. In sub-fraction 8 crystals were formed which were separated and dissolved in hexane to recrystallize. The remaining sub-fractions were not carried out for isolation due to small quantities and left for future work.

#### 5.5.6 Characterization of crystals

The isolated compound was tested for group identification by spraying reagents on a spotted TLC plate. The compound was dissolved in hexane and was applied on a TLC plate using a glass capillary tube. The plate was treated with ceric ammonium nitrate, Dragendorff's reagent, natural product reagent, and anisaldehyde reagent to derivatize the spot. The Salkowski and Liebermann-Burchard tests were also performed. The purity of the compound was assessed by HPLC. The chemical structure was determined by X-ray diffraction crystallography. The structure was further confirmed using UV/Visible spectrum, IR spectrum, and mass spectrometry.

### 5.6 *In vivo* anti-inflammatory screening

#### 5.6.1 Animals and grouping

*Swiss,* male rats, weighing (180–200 g) were acclimatized for 7 days in the Animal Procedure Room for seven days maintaining 12 h light and dark cycle and 22–25 °C room temperature. The rats were segregated randomly into five groups, 6 animals in each. The groups were named groups I, II, III, IV, and V. These groups were labeled as control (untreated and uncompromised), disease control, dexamethasone-treated, CE-treated, and isolated compound-treated, respectively.

#### 5.6.2 Selection and preparation of doses

The extract dose for rats (mg/kg) was calculated by dividing the traditional human dose (5 g/day in divided doses) by 0.162 [36], whereas the dose of Hussainate was predicted based on structural analogy to pentacyclic triterpenoids. So, its dose was considered equivalent to that of the dexamethasone. The extract, Hussainate, and dexamethasone were dissolved in normal saline to prepare the dose.

#### 5.6.3 Dosing and inflammation assessment

The doses were administered orally as group-1 and –II received 1.0 mL/kg normal saline, while group-III, -IV, and -V received 1.0 mg/kg dexamethasone, 500.0 mg/kg CE, and 1.0 mg/kg isolated compound, respectively. After 1 h, the right hind paw of all the rats was observed for inflammation. The rats of group I remained as such whereas 0.1 mL carrageenan suspension (Normal saline containing 1 % carrageenan and 2 % gum acacia) was injected in the sub-plantar region. The volume of the paw was noted at 1 h intervals till 5 h. The reduction in inflammation was calculated using the following equation:

$$\text{Inflammation}\;\text{reduction}\left(\%\right)=\left[1-\frac{\text{dt}}{\text{dc}}\right]x\;100$$

where "dc" represents the change in paw volume of control group and "dt" represents the difference in paw volume of treated group.

#### 5.6.4 Blood and paw tissue collection

After 5 h of carrageenan administration, the blood of each rat was withdrawn from the heat under anesthesia. The blood samples were stored in plain blood collection tubes and used to quantify inflammatory biomarkers. Then, each rat was euthanized by deep anesthesia, and paw tissue was excised. The tissue was blotted and preserved in a 10% formalin solution for histopathology.

#### 5.6.5 Determination of inflammatory markers

RNA was extracted from the blood samples with TRIzol reagent using instructions provided by the manufacturer. A nanodrop 1000 spectrophotometer was used to quantify RNA. A reverse transcriptase kit of Thermo Scientific™, Waltham, USA, was used to synthesize cDNA. SYBR Green Maxima qPCR kit (Thermo Scientific™, Waltham, USA, was used for qPCR analysis. The gene used as a reference was GAPDH. 2-ΔΔCT method was used to determine relative expression. The forward/reverse primers were selected from the published data [37].

#### 5.6.6 Histopathological studies

The paw tissue was dehydrated dipping it in various strengths of aqueous ethanol and absolute ethanol. The dehydrated tissue was fixed in a paraffin block that was used to cut 4–5 μm thick sections. Hematoxylin and eosin were used to stain tissue sections. A light microscope was used to examine the stained sections for pathological changes.

#### 5.6.7 Statistical analysis

One-way ANOVA with LSC post-hoc multiple comparisons was used to compare the means of independent replicates (triplicate/sextuplicate). The given results are the mean along with the standard deviation. The group/set difference was taken as significant if “*p*” was equal to or less than 0.05.

## Ethical approval

The protocol of the study was approved by the Animal Ethical Committee, Punjab University College of Pharmacy, University of the Punjab, Lahore, Pakistan, vide certificate No. AEC/PUCP/1105-A, dated February 10, 2020.

## Funding statement

The funding of any agency is not involved in this paper.

## Data availability statement

The X-ray crystallography data are provided in three files as RES.File, HKL.File and CIF.File. This study is the part of a thesis deposited in the Turnitin Student Paper Repository with ID No. 1866788286, by the Main Library of the University of the Punjab after a plagiarism check. Some data are also as preprint available online at https://assets.researchsquare.com/files/rs-2194713/v1_covered.pdf?c=1667252589.

## CRediT authorship contribution statement

**N. Shehzadi:** Methodology, Data curation. **M Tanveer Khan:** Formal analysis. **S. Zia:** Investigation. **M. Saleem:** Investigation. **S. Akhtar:** Formal analysis. **Farhat Saghir:** Formal analysis. **S. Iftekhar:** Investigation. **A. Mobashar:** Writing – review & editing. **S. Naheed:** Data curation. **N.I. Bukhari:** Supervision, Project administration. **K. Hussain:** Writing – review & editing, Writing – original draft, Supervision, Formal analysis, Conceptualization.

## Declaration of competing interest

The authors declare that they have no known competing financial interests or personal relationships that could have appeared to influence the work reported in this paper.
